# Chamaejasmenin B, a novel candidate, inhibits breast tumor metastasis by rebalancing TGF-beta paradox

**DOI:** 10.18632/oncotarget.10193

**Published:** 2016-06-21

**Authors:** Qi Li, Yajie Wang, Hongbin Xiao, Yujie Li, Xiaoxi Kan, Xiaomin Wang, Ganlin Zhang, Zhixin Wang, Qing Yang, Xi Chen, Xiaogang Weng, Ying Chen, Bingbing Zhou, Yan Guo, Xucen Liu, Xiaoxin Zhu

**Affiliations:** ^1^ Institute of Chinese Materia Medica, China Academy of Chinese Medical Sciences, Beijing, 100700, China; ^2^ Beijing Hospital of TCM, Capital Medical University, Beijing, 100010, China; ^3^ Key Laboratory of Separation Science for Analytical Chemistry, Dalian Institute of Chemical Physics, Chinese Academy of Sciences, Dalian, 116023, China

**Keywords:** Chamaejasmenin B, tumor metastasis, TGF-β paradox, epithelial-mesenchymal transition, tumor microenvironment

## Abstract

Metastasis is the leading lethal factor severely restraining the effectiveness of clinical treatment. TGF-beta is the key regulator for metastasis and influences paradoxically on cancer progression. The known TGF-beta blockers exert little selectivity on its functions, indiscriminately causing the anti-metastatic and pro-growth effects. Under such circumstances, specifically rebalancing the oncological function of TGF-beta provides a crucial oncotarget against metastasis. In our study, we established the screening platform targeting cell motility and identified a potential flavonoid, Chamaejasmenin B (ICJ), extracted from *Stellera chamaejasme* L. It suppressed the migration and invasion in breast cancer cells *in vitro*. Moreover, by dynamical quantification of breast cancer progression in small-animal imaging system, ICJ was proved to be a potent inhibitor of metastasis with minimal toxic side effects. Mechanism study further revealed that ICJ efficiently blocked TGF-beta induced EMT, disrupted the interaction between β3 integrin-TβRII complex and, consequently, resulted in the selective inhibition of FAK:Src:p38 pathway. Meanwhile, specific blockage of this pathway largely attenuated the anti-metastatic function of ICJ. Importantly, in contrast with the antagonistic effects on TGF-beta induced metastasis, ICJ obviously sensitized its cytostatic activity, suggesting that it was not a pan-blocker but a rebalancer for the functional output of TGF-beta. Collectively, by targeting TGF-beta Paradox, we experimentally provided a promising candidate for metastatic intervention.

## INTRODUCTION

Breast cancer is the second killer for women [1–3]. Notably, metastases can be detected in more than 90% of breast cancer patients [4, 5] and about 80% of patients will die of metastasis [6]. Therefore, it is urgent and crucial to develop metastatic-inhibitory drugs for breast cancer. Unfortunately, the effective medicines specifically targeting metastasis remain missing [7, 8].

Recent studies clearly revealed the pro-metastatic effects of epithelial-mesenchymal transition (EMT) [9–12]. EMT is a complicated process featuring the loss of epithelial characteristics and the regain of the properties which can only be observed in mesenchymal cells [13, 14]. Functionally, EMT leads to the re-organization of cytoskeletons and the enhanced cell motility, which facilitate metastasis [5]. Molecularly, EMT highly relies on the alteration of gene expression. This includes the downregulation of epithelial markers like E-cadherin (CDH1) [15, 16] and the upregulation of mesenchymal markers like Vimentin (VIM) [17, 18].

Furthermore, oncological EMT can be primed by TGF-beta, a cytokine playing dichotomous roles in cancers (known as “TGF-beta Paradox”) [19–22]. On one hand, TGF-beta functions as an essential suppressor for tumor growth through the canonical pathway [23]. On the other hand, in the malignant microenvironment, TGF-beta becomes a master stimulator of tumor invasion and metastasis [3, 24]. Recent studies further proved that β3 integrin (ITGB3) is the switching molecule during TGF phenotypic transition. This change implied the counter-activation of the non-canonical TGF-beta signals transduced mainly through the oncogenic pathways such as MAPKs. Therefore, rescuing TGF-beta from tumor promoting to suppressing is an ideal strategy for metastatic intervention [5, 6, 25, 26]]. However, the drugs specifically targeting oncogenic TGF function are still under extensive investigation.

*Stellera chamaejasme* L (SCL) is a perennial herbaceous plant belonging to the Thymelaeaceae family. Although the root of SCL was effective for cancers historically [27, 28], its unspecific toxicity largely limited its application. Leading by this, we prepared serials of SCL extracts and screened their anti-tumor activities systematically. In such work, we also paid more attention to their efficacy-toxicity ratio during treatment. Finally, we have obtained a promising extract named ESC and further isolated a chemical compound Chamaejasmenin B (named ICJ) from it, which had little record of its anti-tumor activities. With minimal toxic effects, we have for the first time identified ICJ as the potent metastatic inhibitor in breast cancer by specifically targeting TβRII: ITGB3: FAK: p38, the central pathway for non-canonical TGF signaling. Notably, different from other TGF pan-antagonists, ICJ was not the universal blocker for TGF-beta. In contrast, the cytostatic effect of TGF-beta can be significantly activated after ICJ treatment, and as such, ICJ re-balanced the functional output of “TGF Paradox” in tumor microenvironment. Our study broke the limit of traditional toxic efficacy of SCL and provided a novel and promising candidate for clinical metastatic intervention.

## RESULTS

### Drug efficacies screening and identification of chamaejasmenin B from SCL

As described in the introduction, TGF-beta is the pivotal oncotarget for controlling of metastasis. Leading by this, we have established the natural products screening platform targeting tumor motility and TGF regulation. During this study, the extracts from *Stellera chamaejasme* L(SCL) greatly attracted our attention.

**Figure 1:**
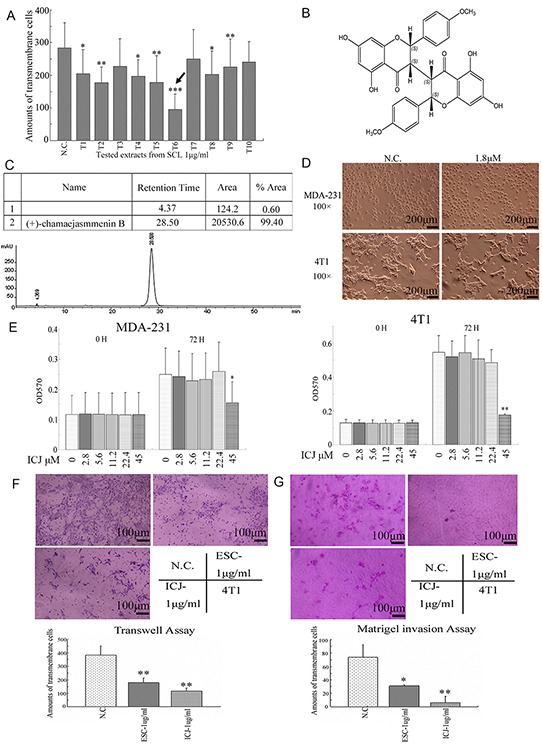
Efficacy screening for SCL extracts and identification of ICJ isolated from ESC **A.** Extracts efficacy screening from SCL targeting tumor cell motility. In this platform, ten different extracts (T1 to T10) with the concentrations of 1μg/ml were prepared by newly established extraction protocol and they were utilized to treated 4T1 for 24 hours. Then the cell motility changes were measured by Transwell assay. As indicated by the black arrow in the figure, one of the extract, T6 (named ESC), possessed the strongest activity against breast cancer migration. **B.** The chemical structure of Chamaejasmenin B (ICJ). The absolute configuration of ICJ was determined through NMR and CD. **C.** Purity detection of prepared ICJ using a WATERS-2695 HPLC equipped with a photodiode array detector (UV wavelength was 295 nm). **D.** The morphological observation of MDA-MB-231 cells (shown as MDA-231 for short) and 4T1 cells treated with 1.8μM (1μg/ml) low-dose ICJ. Images were collected on 24 hours after drug treatment. **E.** MTT assay in breast cancer cells treated with a serial doses of ICJ (from 2.8 to 45μM) for 72 hours. **F.** Transwell assay in ICJ or ESC treated 4T1 cells. Results were further quantified through the cell counting in randomly selected 5 microscopic fields. **G.** Matrigel cell invasion assay in 4T1 cells. The result was quantified through the same method in Figure 1F.

Through efficacy screening, among ten tested extracts, we clearly demonstrated that ESC (named T6) efficiently inhibited breast cancer cell migration at the low dose (Figure 1A). Indicated by this, we further isolated a highly-content compound Chamaejasmenin B (ICJ) from ESC, which had little record of its bioactivity against cancers. Firstly, chemical structure analysis identified that ICJ was a flavonoid with molecular formula of C_32_H_26_O_10_. Its relative molecular mass was 570. The chemical structure of ICJ was showed in Figure 1B and the purity of prepared (+)-chamaejasmenin B was 99.4%, which was determined by the area normalization method using a HPLC equipped with a photodiode array detector (Figure 1C). The purity of the product met the requirement of further pharmacological study.

Next, the dose-toxicity test was performed. According to the result, the cell morphology showed little influence under 1.8μM ICJ treatment (equivalent to 1μg/ml,) in both MDA-MB-231(named MDA-231 for short) and 4T1 high-invasive breast cancer cell lines (Figure 1D). Additionally, the cell proliferation intensity was further quantified by MTT assay. With the same initial cell confluency, after culturing for 72 hours, result showed no significant difference of cell proliferation rate in low-dose ICJ treated group comparing to that in negative control (Figure 1E). From the above data, we could clearly conclude that less than 22.4μM of low-dose ICJ was optimal for drug efficacy studies with little cytotoxicity.

Based on the above results, we next investigated if ICJ, at the non-toxic dose interval, possessed the same efficacy as ESC. As expected, in transwell assay and Matrigel invasion assay, under 1μg/ml ICJ treatment (equivalent to 1.8μM), the transmembrane cells were 179 and 6 respectively, which were more than 3 and 14 times lower than it in negative control. Moreover, ICJ showed stronger activities against cell migration and invasion than ESC, indicating that, in high-invasive breast cancer model, low-dose ICJ had the similar but much higher efficacy as ESC and might be a potent candidate against metastasis (Figure 1F and 1G).

### *In vitro* efficacy identification of low-dose ICJ

**Figure 2:**
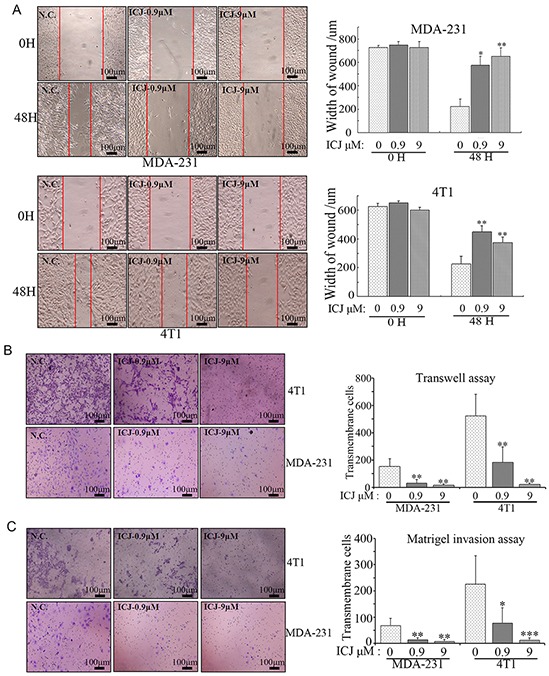
Low-dose ICJ efficiently inhibits cell migration and invasion in breast cancer cells *in vitro* **A.** Wound healing assay in MDA-231 and 4T1 cells. Images were taken on the 0 hour and 48 hours after scratching. The width of the wound was measured to quantify the migration potential in each group. **B.** Transwell assay in MDA-231 and 4T1 cells. Cells were treated with ICJ with the indicated concentrations. Then they were collected and transferred to transwell insert. The cell motility potential was further quantified by the counting of transmembrane cells in 5 randomly selected microscopic fields. **C.** Matrigel invasion assay in MDA-231 and 4T1 cells. The cells were treated for 24 hours and re-paved onto Matrigel-coated transwell insert in 24-well plate. After 18 hours, results were collected through the method described in (B).

Based on the above indication, the detailed efficacy analysis for ICJ was further performed in two high-invasive breast cancer cell lines. Firstly, cell motility was quantified by wound healing assay and transwell assay. In wound healing assay, both cell lines showed high migration potential without ICJ treatment. The wound almost healed in negative control after 48 hours growth. In contrast, the healing ability was significantly and dose-dependently attenuated by ICJ. As shown in Figure 2A, under the same initial scratching width and the same culture time, the final healing width were 600-700μm and 400-500μm in ICJ treated MDA-231 and 4T1 cells respectively, which were about 2-4 times wider than them in negative control groups. Consistently, in transwell assay, ICJ also exhibited potent migration suppressive activity as represented by the 10-15 folded decrease of the transmembrane rate in ICJ treated cells (Figure 2B). The above results clearly revealed the potent efficacy of low-dose ICJ on the inhibition of breast tumor motility. Moreover, the interaction between tumor and extracellular matrix (ECM) is highly dependent on cell proteolytic activity and functions essentially during tumor invasion. Therefore, cell basement membrane matrix (Matrigel) invasive assay was performed. According to the data, ICJ resulted in the 3-6-folded decreases of invasive rates in both MDA-231 and 4T1 cell lines (Figure 2C), strongly suggesting that ICJ blocked the invasive progression of breast cancer *in vitro*.

### *In vivo* identification of anti-metastatic activity of ICJ in breast cancer model

**Figure 3:**
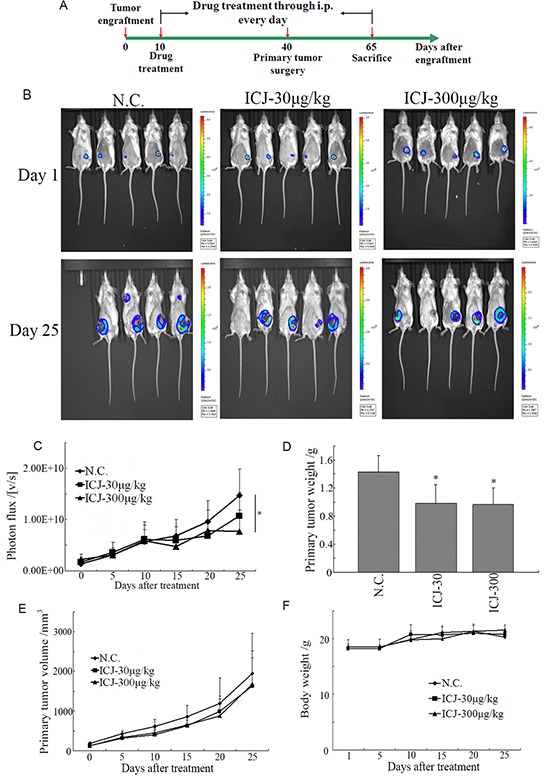
Low-dose ICJ partially inhibits primary tumor growth *in vivo* **A.** The protocol for ICJ efficacy identification *in vivo*. **B.** Detection of primary tumor growth through small animal imaging system. The images shown in the figure were obtained from 5 representative mice and taken on the 1^st^ day or the 25^th^ day respectively. The total photons **C.** primary tumor weight **D.** volume **E.** and body weight **F.** were recorded and statistically analyzed.

To further examine the drug efficacy of ICJ *in vivo*, 4T1 cells stably expressed firefly luciferase (4T1-Luc) were constructed and implanted into the 4^th^ mammary fat pad of female BalB/c mice. Then the tumor bearing mice were administrated with 30 and 300μg/kg ICJ and monitored using a bioluminescence imaging system (Figure 3A).

Firstly, result demonstrated that the growth of primary tumor was partially suppressed as represented by the reduced total photon values in drug treated groups (less than 2-times reduction comparing to negative control) (Figure 3B and 3C). In addition, the tumor weight and volume were also mildly repressed (Figure 3D and 3E). Noteworthy, based on the daily observation, the living conditions of drug exposed mice, reflected by diet status, active intensity or appearance, were not negatively influenced. Accordingly, the body weights of mice in all groups kept at the similar levels and increased with the comparable rates (20~22g) (Figure 3F), indicating that *in vivo* administration of low-dose ICJ induced little toxic side effects in the tumor-bearing mice and these doses were safe and acceptable for our further study.

**Figure 4:**
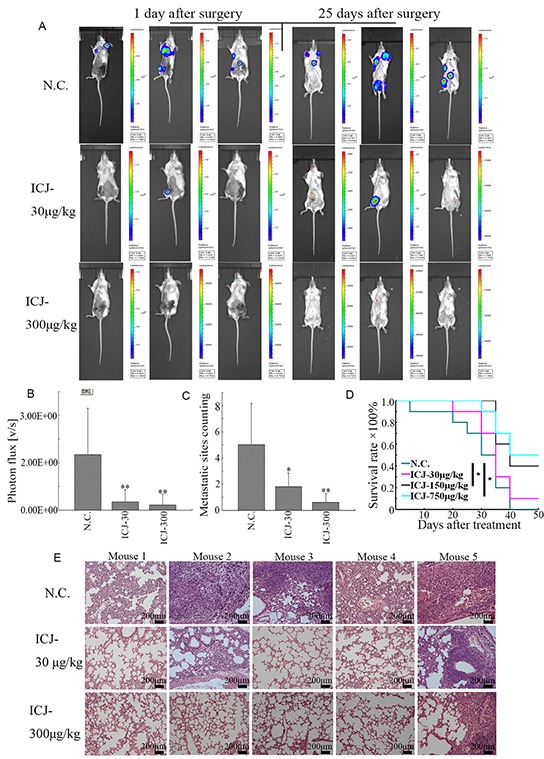
Low-dose ICJ possesses potent metastatic inhibitory activity for breast cancer *in vivo* **A,B.** After surgical removal of primary tumor, the metastatic condition of the animal model described in Figure 3 was monitored by small animal imaging system. The images from 3 representative mice were shown in (A). Images were quantified by measuring the total photon values of metastases (B) and the counting of metastatic sites (C). **D.** Survival analysis of the ICJ treated tumor-bearing mice. The survival period has been obviously prolonged by ICJ. **E.** Histological analysis of the metastatic intensity in lungs from the mice mentioned above. The lung tissue samples were randomly selected from 5 mice in different groups.

More importantly, to dynamically trace the metastasis of breast cancer, primary tumors were removed by mastectomy on the 30^th^ day of drug treatment. After surgery, the metastatic sites can be fully visualized and the difference of metastatic conditions between the negative control and ICJ exposed groups can be specifically observed. On the 1^st^ day after the surgery, the metastases have already well established and widely distributed in negative control mice. In contrast, the metastatic images can rarely be detected in ICJ exposed mice. Of note, after another 25 days of growth, the metastases still can not be imaged under ICJ treatment (Figure 4A). Based on such indication, the total photon values of metastases were then calculated and the metastatic sites were counted. In comparison with the primary tumors, the values of total photon from the metastatic sites were statistically and dramatically decreased in responses to low-dose ICJ treatment (Figure 4A and 4B). The average of total photon value in ICJ treated mice was 1.12×10^9^, which was 8.14 times lower than it in negative control (9.12×10^9^). Accordingly, the number of metastatic sites was dose-dependently decreased in the presence of ICJ (Figure 4C). Notably, as analyzed by the survival curve, ICJ could effectively alleviate the disease progression and reduce the mortality of breast cancer (Figure 4D).

In addition, pathological analysis of the lungs from tumor bearing mice showed that the normal tissue structure in lungs was severely damaged by malignant colonization in negative control mice. In contrast, ICJ greatly prevented such pathological changes and protected the lungs against metastasis. This data histologically provided another evidence for our study (Figure 4E). Taken together, in addition to its mildly inhibitory effects on primary tumors, our results revealed the powerful and specific suppressive efficacy of ICJ targeting breast cancer metastasis (especially for pulmonary metastasis) *in vivo*.

### The antagonistic effects of ICJ on TGF-beta induced EMT in breast cancer cells

**Figure 5:**
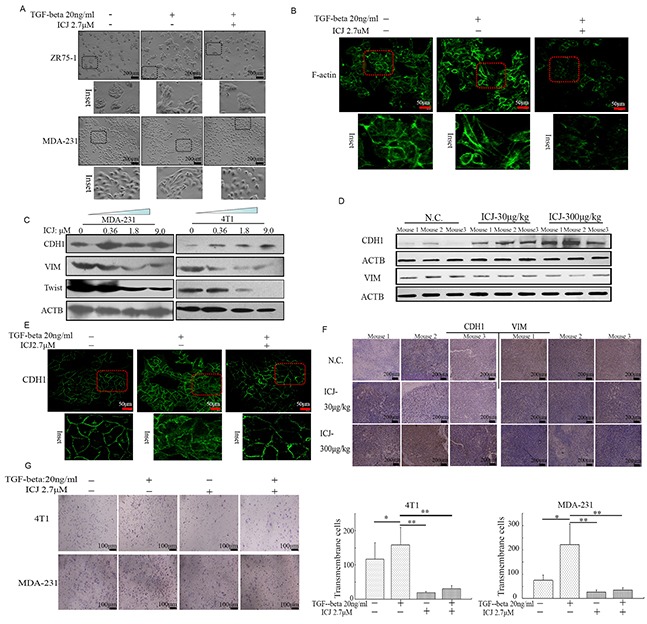
Low-dose ICJ blocks TGF-beta induced EMT in breast cancer cells **A.** Morphological observation in ICJ treated breast cancer cells in the absence or presence of TGF-beta. After 48 hours of treatment, light-microscopic images were collected. Inset showed the details of the cellular morphological features. **B.** Confocal microscopic observation of the subcellular localization of F-actin in MDA-231 cells. The images showed the reduced polymerization and the re-organization of actin in response to ICJ treatment. **C.** Detection of EMT related markers in cells treated with ICJ (from 0.36 to 9.0μM) for 48 hours. **D.** Western blot verification of EMT-related markers by using the primary tumors (3 mice/group) obtained from the *in vivo* experiments mentioned in Figure 3. **E.** E-cadherin (CDH1) subcellular localization through Immuno-fluorescence analysis. The cells were treated with ICJ for 48 hours and CDH1 was visualized with FITC-labeled antibody. The nuclei were labeled by DAPI. **F.** Immunohistologicalchemical analysis of EMT molecular markers in primary tumors. The tissue samples were randomly selected from 3 mice in each group. **G.** Matrigel invasion assay of cells treated with ICJ alone or combined treated with ICJ and TGF-beta for 24 hours. The amounts of transmembrane cells were further quantified in five randomly selected microscopic fields.

Theoretically, the acquisition of metastatic potential in breast tumors is positively associated with the oncological activation of EMT. Additionally, TGF-beta is widely accepted to be the potent inducer for EMT and could be used to construct the experimental EMT model for pharmacological research [5, 6]. Taking this into account, we made efforts to reveal the potential correlation between ICJ and EMT in TGF-beta treated model. Firstly, microscopic observation demonstrated that TGF-beta successfully induced the mesenchymal changes characterized by the elongated fibroblastoid-like shape and loss of cell-cell contact in breast cancer cells. However, low-dose ICJ effectively reversed these changes and restored the epithelial features which were the cobblestone-shape and the enhanced tight junctions between cells (Figure 5A). The results morphologically indicated that ICJ may act as an antagonist of TGF-beta. Next, the subcellular localization and the polymerization of actin, the indicative parameters for metastasis [29], were further observed by confocal microscopy. The data demonstrated that TGF-beta readily induced the cytoskeleton re-organization, featuring the strengthened cortical distribution and the disordered organization of the actin stress fibers. However, actin polymerization was remarkably weakened by ICJ (Figure 5B).

In parallel with the morphological changes, the molecular markers for EMT were also detected. Data demonstrated that the tumor suppressor E-cadherin (CDH1), which is also the hallmark for epithelial cells [30], was dramatically increased in ICJ treated cells. In contrast, the mesenchymal marker Vimentin (VIM) and the EMT-initiating factor Twist were downregulated (Figure 5C). This molecular expression pattern can also be detected in the primary tumor samples *in vivo* (Figure 5D). We next assessed the subcellular localization of CDH1. Consistent with the EMT blocking effect, the distribution of CDH1 was obviously recruited from cytoplasm to the membrane by TGF-beta and ICJ combined treatment (Figure 5E). In addition, through histological detection, IHC analysis proved that low-dose ICJ re-established the epithelial-mesenchymal polarization in primary breast tumors (Figure 5F).

Moreover, Matrigel assay showed that the percentage of transmembrane cells was statistically reduced in ICJ and TGF-beta combined treated group, functionally suggesting that ICJ may reverse the pro-invasive effect of TGF-beta (Figure 5G). Collectively, our results demonstrated that ICJ may efficiently block EMT and invert the phenotype transition stimulated by TGF-beta in breast cancer cells.

### Selective inhibitory effects of ICJ on TGF functional output

**Figure 6:**
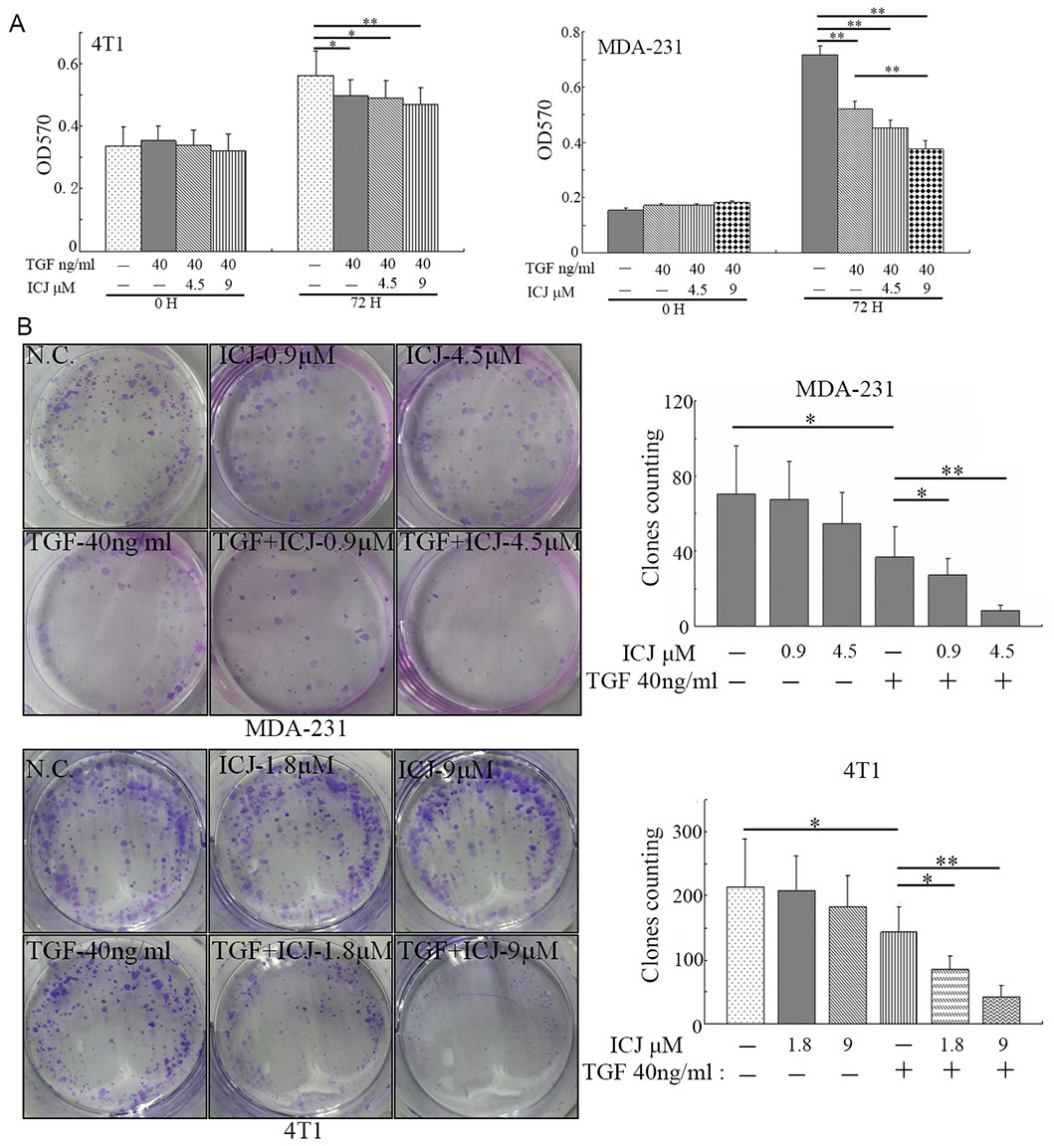
Low-dose ICJ synergizes with TGF-beta to inhibit tumor proliferation **A.** MTT assay in ICJ and TGF-beta combined treated MDA-231 and 4T1 cells. Optical density values of 570nm (OD570) were measured on the 0 or 72 hours respectively. **B.** Colony formation assay in ICJ and TGF-beta combined treated cells. 500 cells were paved onto 6-well plate and treated with ICJ or TGF. Drug containing medium was changed every 3 days. Then the cells were stained on the 15^th^ day after drug treatment and the amounts of clones in each well were statistically calculated.

Based on our study, we have proved that ICJ targeted TGF-beta and blocked its oncogenic function. Due to the duality of TGF-beta during cancer progression, we further functionally detected the influence of ICJ on its tumor-inhibiting effects. Through MTT assay in 4T1 cells, ICJ selectively preserved the cytostatic effects of TGF-beta. More importantly, in MDA-231 cells, we even observed the proliferation was significantly decreased in ICJ and TGF-beta combined treated groups compared with either negative control or the TGF-beta single treated group. These results indicated the synergistic association between ICJ and TGF-beta in tumor growth inhibition (Figure 6A). Consistently, in colony formation assay, we also observed the reduced colony formation potential in response to combined treatment. The highest inhibitory rate was 91.8% in MDA-231 cells and 84.1% in 4T1 cells, which were 2.12 and 2.35 times higher than them in cells treated with TGF-beta alone (Figure 6B). Collectively, our results revealed that ICJ had little inhibitory activity or even sensitized the cytostatic effect of TGF-beta in cancer cells. This observation suggested that, instead of the unselective inhibition of TGF-beta, ICJ induced the functional shift of TGF-beta toward the cytostatic direction in malignant microenvironment.

### Regulation of ICJ on TGF-beta Paradox

**Figure 7:**
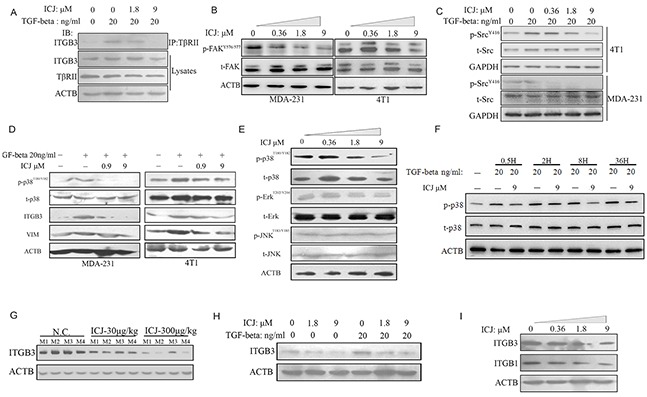
Low-dose ICJ targets non-canonical signaling of TGF-beta **A.** Co-immunoprecipitation analysis in MDA-231 cells. Cells were pre-treated with ICJ for 36 hours and stimulated by TGF-beta for 15 minutes. IP: Immunoprecipitation; IB: Immunoblotting; Lysates: Whole cell lysate control. **B.** Western blot analysis of FAK phosphorylation in ICJ treated MDA-231 and 4T1 cells for 36 hours. **C.** Detection of Src phosphorylation in ICJ treated and TGF-beta stimulated MDA-231 and 4T1 cells. **D, E.** Detection of the phosphorylation of MAPK members in ICJ treated breast cancer cells for 36 hours. **F.** Time-course detection of p-38 phosphorylation level in response to ICJ and TGF co-treatment. The p38 phosphorylation was monitored from 0.5 to 36 hours after treatment in 4T1 cells. Result revealed that ICJ could inhibit p38 pathway in a wide time-window. **G.** Detection of the ITGB3 expression in primary tumor samples from experiment mentioned in Figure 3. Four mice samples were randomly selected in each group (named M1 to M4). **H.** Detection of ITGB3 expression in TGF-beta and ICJ combined treated MDA-231 cells for 36 hours. **I.** *In vitro* analysis of ITGB1 and ITGB3 expression in MDA-231 cells.

Inspired by the blockage effects of ICJ on TGF-beta, we intended to further reveal its underlying mechanism in breast cancer. Recent study has proved that, by mediating the signal of TGF-beta to MAPKs, β3 Integrin (ITGB3) is recognized as the switching molecule during the phenotypic conversion of TGF-beta from a tumor suppressor to a promoter [31]. More importantly, the formation of ITGB3-TβRII complex is the symbolic event for the activation of oncogenic TGF-beta signal pathway. Therefore, we firstly detected the physical interaction between ITGB3 and TβRII by co-immunoprecipitation assay. In this study, to avoid the expression influence of ITGB3 after long-term TGF-beta induction, ICJ treated cells were transiently stimulated by TGF-beta for 15 minutes. Under such setting, endogenous expression of ITGB3 maintained at the similar level among all groups as identified by western blot analysis. Under such circumstances, results clearly revealed that TGF-beta successfully enhanced the binding between ITGB3 and TβRII. Conversely, ICJ could effectively disrupt this binding (Figure 7A); initially predicting that, by targeting ITGB3-TβRII complex, ICJ selectively blocked the oncogenic TGF-beta signaling.

To further reveal the inhibition effects of ICJ on the blockage of the ITGB3-TβRII complex, we found that, in TGF-beta signal transduction cascade, Focal Adhesion Kinase (FAK) was the mediator for the ITGB3-TβRII interaction and aberrant activation of FAK was required for the initiation of protumoral TGF-beta signal [26]. Indicated by this, the phosphorylation level of FAK was detected. Result revealed that ICJ efficiently inhibited the activation of FAK in both two breast cancer cell lines (Figure 7B).On the basis of this result, we further found that, in the TGF-beta non-canonical signal pathway, Src is proved to be the essential molecule responsible for bridging the interaction between TβRII and MAPKs. We therefore detected the Src phosphorylation in the presence of ICJ and TGF-beta. Result showed that the Src phosphorylation level was efficiently inhibited by the cotreatment of TGF-beta and ICJ (Figure 7C). This result provided another evidence for the inhibition effect of ICJ in the oncological TGF-beta signal transduction.

Because the pro-metastatic effects of TGF-beta are dependent on the activation of MAPKs [32], in the next study, we examined the phosphorylation levels of its members, including p38, ErK and JNK. Result showed that, ICJ specifically attenuated the phosphorylation level of p38 in a dose-dependent manner; whereas, it had little impact on the JNK and Erk activation. Furthermore, in the time-course study, by cotreatment of TGF-beta and ICJ for a serial of times, we also revealed that ICJ could prevent the p38 activation in a wide time-window. Collectively, our results suggested that ICJ might directly and specifically block p38 pathway in response to TGF-beta and in turn, rebalance the canonical and non-canonical signaling in breast cancer cells. (Figure 7D to 7F).

Finally, based on other reports, the phenotypic transition of TGF-beta and the enhancement of tumor invasion and metastasis are coincided with the upregulation of ITGB3. Additionally, inhibition of ITGB3 could functionally abolish the pro-malignant changes in breast cancer cells. Therefore, the expression change of ITGB3 was detected in ICJ exposed primary breast tumors. In this assay, we randomly selected 4 mice tumor samples from each group. As expected, ITGB3 was dose dependently downregulated by ICJ *in vivo* (Figure 7G). We further detected the influence of ICJ on ITGB3 expression in the presence of TGF-beta. As shown in the Figure 7H, TGF-beta successfully stimulated the ITGB3 expression and ICJ significantly reversed such changes. Additionally, the compensatory response between ITGB3 and ITGB1 (β1 Integrin) is believed the main resistant mechanism in TGF-beta targeted treatment and the co-inactivation of these two molecules is necessary for the efficient suppression of metastasis [33]. We therefore tested the expression level of ITGB1 by Western blot. Result demonstrated that, similar to the change of ITGB3, ITGB1 was also obviously inhibited by ICJ, (Figure 7I). Therefore, our results demonstrated that ICJ was the dual inhibitor for both ITGB1 and ITGB3, which effectively prevented the compensatory restoration of TGF-beta caused oncogenic EMT.

Collectively, our evidence indicated that ICJ functioned as the p38 deactivator and the ITGB3 suppressor mainly through the disassociation of TβRII-ITGB3 complex. Based on such mechanism, ICJ may specifically block the TGF non-canonical pathway. At the functional level, it selectively inhibited the pro-metastatic activities of TGF-beta and, consequently, reestablished the balance between canonical and non-canonical signaling to enhance its cytostatic function in breast cancer cells.

**Figure 8:**
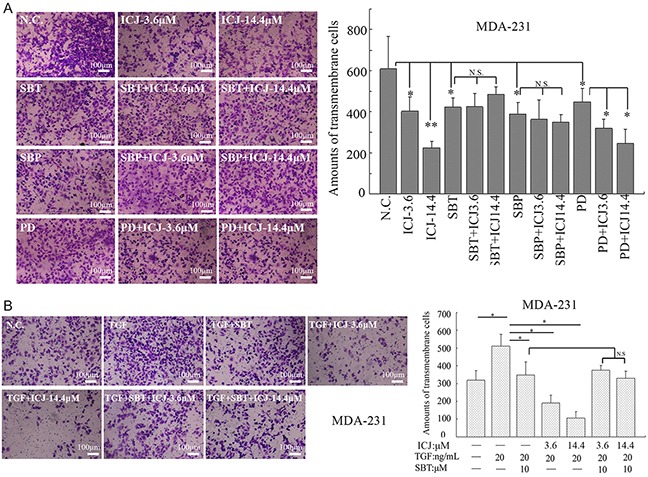
The anti-metastatic activity of ICJ is dependent on the activation of non-cannonical TGF-beta pathway **A.** Transwell assay in MDA-231 cells treated with ICJ and small molecular inhibitors targeting TβRII (SBT), p38 (SBP) and Erk (PD). The prestarved cells were treated with ICJ and the blockers for 24 hours. **B.** Transwell assay in MDA-231 cells in the presence of TGF-beta. Cells were pre-starved and treated for 24 hours with 3.6, 14.4μM of ICJ and 20ng/ml of TGF-beta either in the absence or presence of SBT. The result showed that the cell motility inhibition by ICJ was largely dependent on the functional completeness of TGF-beta.

Next, to further verify whether the anti-metastatic activity of ICJ relied on the activation of TGF: p38 pathway, TGF-beta inhibitor (SB431542, SBT), p38 inhibitor (SB203580, SBP) and Erk inhibitor (PD98059, PD) were used. The cell motility was evaluated in the presence of these three inhibitors. In transwell assay, we clearly observed that the anti-metastatic efficacy of ICJ was abrogated specifically in TGF-beta and p38 deficient cells. In contrast, Erk inhibition failed to reverse the impaired migration potential induced by ICJ (Figure 8A). Besides, in the presence of TGF-beta, the transmembrane cells were obviously increased and ICJ markedly reversed this trend in a dose-dependent manner. However, the transmembrane cells in ICJ and SBT combined treated groups were kept at the approximate levels compared with the SBT solely treated group (Figure 8B). This result proved that the motility inhibitory efficacy of ICJ cannot be detected under the TGF signaling-blocked condition. Its dose dependency was also attenuated in the presence of SBT. Taken together, our results suggested that the TβRII: p38 non-canonical signaling was required for the anti-metastatic activity of ICJ.

## DISCUSSION

Medical herb *Stellera chamaejasme* L. has been used as the anti-tumor drug for thousands of years in Chinese history. However, traditional concept simply held the view that the tumoricidical efficacies of SCL highly relied on its cytotoxicity, and as such, the side effects have become the leading obstacles for its clinical application. Through our efforts, we have firstly identified a potential compound, ICJ from SCL, which inhibited breast tumor metastasis with little toxic side effects. Our results provided a novel strategy for SCL during cancer treatment which may largely avoid the adverse effects, ensuring the safety of drug treatment. This will further expand the clinical application of ICJ in the future.

Regulation of tumor progress mediated by TGF-beta is highly complex and contradictory [34]. Based on such “Paradox theory”, the key to use TGF-beta as the treatment oncotarget is to reestablish its pathological balance in tumor microenvironment [25]. This offers a practical approach to alleviate metastasis without adverse effects [25, 35–38]. However, till now, most of the known TGF inhibitors are pan-antagonists which block TGF functions unspecifically and, as a result, the current anti-TGF strategies are shown to promote the early growth of breast cancer in clinical trials [25]. Delightfully, our results demonstrated that ICJ was not simply a broad-spectrum TGF-beta blocker. By the efficient disassociation of ITGB3-TβRII signal-initiating complex, ICJ could suppress tumor metastasis while simultaneously had little influence on the activation of pro-apoptotic effects of TGF-beta. Therefore, in contrast to the known antagonists, ICJ demonstrated clear selectivity in reshaping the balance of “TGF-beta Paradox” and shifting its functions toward the direction of cytostasis in malignant cells.

Taken together, we have reasons to believe that ICJ is the promising candidate specifically targeting metastasis. Our studies might make it possible to develop combined treatment of ICJ with the classic tumor-toxic drugs such as Paclitaxel. However, the detailed mechanism of ICJ is still not fully understood. Particularly, the most upstream events mediating ICJ efficacy and the molecular mechanism for ITGB3 regulation need more evidence to be clarified. Moreover, for the future clinical application, the efficacy-based chemical modification, the pharmacokinetic identification and the short-term and long-term toxicological test are extremely needed.

## MATERIALS AND METHODS

### Reagents and cell culture

The isolation protocol for ICJ is summarized as following: the 90% EtOH-H_2_O elution fraction (3g) from ESC was firstly dissolve in DMSO and separated by preparative HPLC (75% MeOH-H_2_O, volume concentration, containing 0.2 % formic acid), resulting the purification of (+)-chamaejasmenin B (300mg). The monitoring UV wavelength was 295nm and the corresponding retention time was about 9.4 minutes. The absolute configuration of ICJ was determined through NMR and CD and the purity detection of prepared ICJ was performed using a WATERS-2695 HPLC equipped with a photodiode array detector (UV wavelength was 295 nm).

Recombinant Human TGF-beta1 was purchased from Peprotech Corporation (100-21, New Jersey, USA). The preparation of TGF-beta1 solution was strictly performed according to the manufacture's instruction; D-luciferin Firefly potassium salt was purchased from Gold Biotechnology, Inc (St. Louis, MO); Primary antibodies of Twist, phospho-FAK (Tyr576/577), total-FAK, were purchased from Santa Cruz Biotechnology, Inc. (California, USA); Primary antibodies of ITGB3, phospho-p44/42 (Thr202/Tyr204), phospho-JNK (Thr183/Tyr185), phospho-p38 (Thr180/Tyr182), total p42/p44, total-JNK, total p38, CDH1, VIM, were purchased from Cell signaling Technology, Inc. (Massachusetts, USA). DMEM, RPMI1640, Fetal bovine serum was purchased from Invitrogen (Carlsbad, CA, USA). 4,6-diamidino-2-phenylindole (DAPI) and 3-(4,5-dimethylthiazol-2-yl)-2,5-diphenyltetrazolium bro-mide (MTT) was purchased from Sigma (USA).

Breast cancer cell lines, MDA-MB-231 (named MDA-231 for short), ZR75-1 and 4T1, were purchased from American Typical Collection Center (Maryland, USA) and were maintained in RPMI-1640 medium (Gibco, C11875500bt) supplemented with 10% (v/v) fetal bovine serum (Hyclone, SH30070) and 1% Pen/Strep (v/v) at humid 37°C incubator with 5% CO_2_.

### Wound healing assay

5×10^5^ Cells were paved into 6-well plates and cultured to 90% confluency. Then scratch the cell layer with a micropipette tip (10μl) and wash each well twice with PBS to remove the suspended cells. The pictures of wounded monolayers were taken at the indicated time points after wounding and the width of each wound was measured to quantify the cell motility.

### Small animal imaging

4T1 cells with stable firefly luciferase expression were provided by Caliper Life Sciences, (Hopkinton, MA) to trace the metastasis *in vivo*. In this assay, 1×10^4^ cells were resuspended in PBS and inject into mammary fat-pads of 8-week old female BALB/c mice. The tumor-bearing mice were randomly grouped (10 mice per group) on the 10^th^ day after tumor transplantation. Subsequently, the mice were intraperitoneally injected with ICJ everyday till to the end of the experiment. The body weights and the tumor sizes (calculated by the ellipsoid formula: volume = 1/2 × length × width^2^) were regularly measured. On the 30^th^ day after drug administration, the primary tumors were surgically removed and the mice were continuously monitored for another 25 days. Animals were sacrificed on the 65^th^ day after tumor transplantation. All procedures were conducted in accordance with the China Experimental Animal Ethics Committee.

The primary tumor growth and the metastatic progression were dynamically visualized and quantified through IVIS Spectrum Imaging System (Caliper Life Sciences, Hopkinton, MA). Before detection, 200μl D-firefly potassium salt (reconstitution in sterile PBS to the concentration of 15mg/ml) was used through hypodermic injection. After 10 minutes, the mice were anaesthetized by isoflurane and imaged by the small animal imaging system.

### Transwell cell migration and matrigel cell invasive assay

Breast cancer cells were serum starved for 24 hours and 1×10^5^ cells were seeded onto the Transwell chamber (8 μm pore size, BD Falcon, New Jersey, USA). For cell invasive assay, the Transwell chamber was precoated with basement matrix (Matrigel, BD, New Jersey, USA). Then, the inserts were placed into a 24-well plate. The upper chamber contained serum-free medium and the medium with 10% FBS was added into the lower chamber. After culturing for 18 hours, gently swab the cells on the top surface of the upper chamber and fixed with 4% paraformaldehyde for 10 minutes. Following staining of the membrane with crystal violet (0.1% in ethanol), the transmembrane cells were imaged and further quantified by counting 5 randomly selected fields under light microscope (200× magnification).

### Cell proliferation assay

In Colony formation assay, 5×10^2^ cells were seeded onto 6-well plate. After culturing for 24 hours, drugs were added and the cells were cultured for 15 days. Then the clones were fixed and visualized by crystal violet. The amounts of independent clones (clone diameter ≥ 0.5 mm) were further quantified.

In MTT assay, the cells were paved onto 96-well plates (3000 cells/well). The drugs were added into each well on the 24 hours after pavement. At the indicated time points, 100μl MTT was added and reacted for 4 hours. Following dissolved in DMSO, the optical density value in 570nm (OD570) was detected.

### Western blot analysis

Cells were seeded onto 6-well plates (2×10^5^ cells/well). On the indicated time points, the cells were harvested and an appropriate volume of lysis buffer (20 mM Tris HCl, pH 7.4, 150 mM NaCl, 1 mM EDTA,1mM EGTA, 1 mM PMSF and 1% Triton X-100) was added. Through BCA protein quantification (Pierce, USA), the same amounts of denatured protein samples were separated by sodium dodecyl sulfate-polyacrylamide gel electrophoresis (SDS-PAGE) and the gels were electrically blotted onto NC membrane (Amersham Pharmacia, UK). After blocking with 5% BSA solution, the membrane was incubated overnight with the primary antibodies at 4°C. Then the membrane was washed for 3 times by tris-buffered saline buffer with tween (TBS-T) and incubated with secondary antibody conjugated with HRP. The aim bands were visualized using chemiluminescence detection reagents (Thermo, USA). β-actin (ACTB) was loaded as the internal control.

### Statistical analysis

The results shown in each figure were expressed as arithmetic means ± SD. *P* value lower than 0.05 represents the statistical significance. All experiments were performed at least triplicate and statistically analyzed by SPSS (17.0). One-way analysis of variance (one-way ANOVA) with LSD and Tukey's post hoc test was using for each quantification.* *P*<0.05; ** *P*<0.01; *** *P*<0.001

## Abbreviations

EMT: Epithelial-Mesenchymal Transition
ESC: Extract from Stellera chamaejasme L
ICJ: Chamaejasmenin B
SCL: Stellera chamaejasme L
